# Perspective: Cognitive Behavioral Therapy for Insomnia Is a Promising Intervention for Mild Traumatic Brain Injury

**DOI:** 10.3389/fneur.2020.530273

**Published:** 2020-10-07

**Authors:** Jessica R. Dietch, Ansgar J. Furst

**Affiliations:** ^1^War Related Illness and Injury Study Center (WRIISC CA), VA Palo Alto Health Care System, Palo Alto, CA, United States; ^2^Department of Psychiatry and Behavioral Sciences, Stanford University School of Medicine, Palo Alto, CA, United States; ^3^Department of Neurology and Neurological Sciences, Stanford University School of Medicine, Palo Alto, CA, United States; ^4^Polytrauma System of Care (PSC), VA Palo Alto Health Care System, Palo Alto, CA, United States

**Keywords:** CBT (cognitive-behavioral therapy), insomnia, traumatic brain injury, mild traumatic brain injury (mTBI), sleep, cognitive behavioral therapy for insomnia (CBT-I)

## Abstract

Mild traumatic brain injury (mTBI) is a significant public health problem. Insomnia is one of the most common symptoms of TBI, occurring in 30–50% of patients with TBI, and is more frequently reported in patients with mild as opposed to moderate or severe TBI. Although insomnia may be precipitated by mTBI, it is unlikely to subside on its own without specific treatment even after symptoms of mTBI reduce or remit. Insomnia is a novel, highly modifiable treatment target in mTBI, treatment of which has the potential to make broad positive impacts on the symptoms and recovery following brain injury. Cognitive-behavioral therapy for insomnia (CBT-I) is the front-line intervention for insomnia and has demonstrated effectiveness across clinical trials; between 70 and 80% of patients with insomnia experience enduring benefit from CBT-I and about 50% experience clinical remission. Examining an existing model of the development of insomnia in the context of mTBI suggests CBT-I may be effective for insomnia initiated or exacerbated by sustaining a mTBI, but this hypothesis has yet to be tested via clinical trial. Thus, more research supporting the use of CBT-I in special populations such as mTBI is warranted. The current paper provides a background on existing evidence for using CBT-I in the context of TBI, raises key challenges, and suggests considerations for future directions including need for increased screening and assessment of sleep disorders in the context of TBI, examining efficacy of CBT-I in TBI, and exploring factors that impact dissemination and delivery of CBT-I in TBI.

## Background

Mild traumatic brain injury (mTBI) is a significant public health problem for many populations including children, adolescents, young adults, older adults, athletes, and military personnel. mTBI is associated with numerous negative sequalae including mental and physical health, work and social functioning, and financial burden to the injured individual and society. Up to 80% of the 1.7 million traumatic brain injuries (TBI) in America each year are classified as mild (1). Despite the prevalence of mTBI, no compelling treatments exist. More work exploring treatment targets in mTBI is a high priority. mTBI commonly precipitates or exacerbates sleep disturbances which can in turn worsen other symptoms or sequelae of mTBI (e.g., fatigue, depression, pain) and interfere with both neural and physical rehabilitation (2). Insomnia, the most common sleep disturbance following mTBI, is a largely unexplored and highly modifiable intervention target for improving rehabilitation and long-term outcomes in mTBI and thus an important area of focus for future research.

## Insomnia in TBI

Untreated insomnia is associated with numerous mental and physical health sequelae (3), increased healthcare utilization (4), work problems (5), and financial burden on both the individual and society (6). Insomnia is one of the most common symptoms of TBI; 30–50% of patients report insomnia that developed or worsened following TBI (7) which is substantially elevated above general population prevalence [i.e., 6–10%; (8, 9)]. Insomnia can develop immediately following the TBI or later during the recovery period and is unlikely to remit without targeted treatment even after TBI symptoms reduce or remit (10). Insomnia disorder is characterized by disturbances in both sleep (i.e., difficulty initiating or maintaining sleep) and wake (e.g., fatigue, difficulty concentrating) domains which, paired together, cause clinically significant distress or impairment (11). Counter to expectations, insomnia is reported at a higher rate in individuals with mTBI compared to moderate or severe TBI (12–15). Reasons for this apparent paradox are not entirely clear, but individuals with mTBI may be more likely to notice or report sleep disturbances compared to individuals with more severe TBI who are coping with greater impairments in memory and cognitive functioning and may have decreased self-awareness (15). Many symptoms of mTBI and insomnia overlap, including fatigue, poor attention and concentration, irritability and poor mood, and memory difficulties. Although insomnia has traditionally been conceptualized as a symptom secondary to a primary disorder (e.g., depression), recent thinking suggests insomnia takes on a life of its own and warrants separate, targeted clinical focus (16). Because of the global impact of insomnia, successful treatment has the potential to substantially improve symptoms and recovery following mTBI.

## Cognitive-Behavioral Therapy for Insomnia

Cognitive-behavioral therapy for insomnia (CBT-I) is efficacious across many populations (17–23) and thus is the first-line treatment for insomnia as recognized by multiple organizations including the American College of Physicians (24), European Sleep Research Society (25), American Academy of Sleep Medicine (26), NIH Consensus State-of-the-Science Conference (27), and VA/DoD clinical practice guidelines (28). Numerous clinical trials and systematic reviews demonstrated between 70 and 80% of patients with insomnia experience enduring benefit from CBT-I and about 50% experience clinical remission (29). CBT-I typically includes multiple cognitive and behavioral treatment components, most commonly sleep restriction, stimulus control, relaxation training, cognitive therapy, and psychoeducation/sleep hygiene (29). Taken together, CBT-I reduces wakefulness and promotes sleep in the bed and bedroom, regularizes the sleep/wake cycle, reduces sleep-related anxiety and arousal, and reshapes unhelpful thoughts about sleep. A typical course of CBT-I is delivered to a patient across 4–8 weekly sessions although many alternative formats and delivery strategies have demonstrated efficacy (e.g., internet-delivered, group therapy, abbreviated treatment). Evidence suggests in addition to clinical psychologists and psychiatrists, nurse practitioners (30) and masters-level clinicians (31) can be trained to deliver CBT-I effectively making it a versatile, flexible intervention. However, uptake of CBT-I is low due to numerous barriers including limited patient and provider awareness of the intervention and lack of trained providers (32). It is difficult to ascertain the exact number of individuals competently trained to delivery CBT-I, although existing data suggests a paucity of trained providers. There are currently only 106 providers certified in the provision of Behavioral Sleep Medicine [which encompasses CBT-I; (33)]. Additionally, per a 2016 report 598 mental health providers completed training in CBT-I via the Veterans' Administration rollout (34). It is likely that many more providers (e.g., psychologists) deliver CBT-I, but the amount and quality of services provided is unknown. Judging from anecdotal reports of high referral volume and extensive waitlists within clinics providing behavioral sleep medicine services, it is likely that the current demand for CBT-I exceeds capacity. Thus, providers embedded in settings with a high volume of patients with mTBI may consider seeking training in CBT-I (e.g., www.cbtiweb.org) to help alleviate this demand.

## Pharmacological Treatment of Insomnia in mTBI

CBT-I is typically preferred over pharmacological interventions for insomnia (i.e., hypnotics) because in comparison it has consistent long-term benefit and a minimal side effect profile [for a review of pharmacological interventions for insomnia in TBI, see (35)]. Side effects of common hypnotics (e.g., benzodiazepines, non-benzodiazepine receptor agonists) are particularly concerning for patients with mTBI given overlap with symptoms of mTBI including daytime sleepiness, fatigue, dizziness, headache, mental slowing, and attentional and memory difficulties. Further, emerging evidence suggests long-term use of hypnotics may be associated with increased risk of dementia (36–38), particularly for TBI patients (39), and mortality (40, 41). Given the potential risks of hypnotics, CBT-I is a clear first choice for treatment of insomnia in patients with mTBI, although they need not be mutually exclusive treatments and can in some circumstances be used together effectively.

## A Conceptual Model of the Development of Insomnia in the Context of mTBI

The behavioral or “3P” model of insomnia posits three primary factors which contribute to the development and maintenance of chronic insomnia over time: predisposing, precipitating, and perpetuating factors (42). The 3P model provides some insight as to how CBT-I could be particularly beneficial to an individual with co-occurring mTBI and insomnia. The first factor of the model, termed “predisposing,” includes elements which inherently put an individual at increased risk for insomnia (e.g., genetics, personality, chronotype). For a given individual with mTBI, prior history of insomnia and prior head injuries are likely elements which could contribute to a predisposition to insomnia. Although predisposition may increase risk for insomnia, it generally does not produce insomnia at the clinical level in the absence of the other two factors (42). The second factor, “precipitating event,” suggests in most cases an identifiable event or set of events (e.g., military service, illness, sports injury) contributes to the development of insomnia in the acute phase (i.e., <1 month). Certainly, a mTBI could serve as the event precipitating a bout of insomnia; many acute changes following mTBI such as physical and/or neurological changes, pain, psychological difficulties (e.g., depression, posttraumatic stress), and changes in activity level are commonly associated with the development of insomnia (10). However, insomnia is unlikely to persist into the recovery period (e.g., after mTBI-related changes subside or adjustment to stressors occurs) in the absence of the third factor (42).

The third factor of the behavioral model of insomnia, “perpetuating,” refers to the tendency of individuals with insomnia in the acute phase to make changes to their behaviors and thoughts related to sleep in order to attempt to cope with the development of sleep disturbances (42). For example, a patient with mTBI who subsequently developed insomnia might attempt to go to bed early, sleep in late, or take daytime naps to “make up for” sleep lost due to insomnia. During the acute phase following mTBI, patients can experience a period of increased sleep need (i.e., pleiosomnia) above the pre-injury baseline (43), which may be followed by an eventual return to baseline sleep duration, although this phenomenon may be more common or severe among individuals with pain (44) or with more severe injuries (45). However, the patient may continue to attempt to sleep longer (i.e., spend more time in bed) even after they are no longer able to produce sleep at the increased quantity. This will result in extended periods of wakefulness in the bedroom environment, which decreases the association between the bedroom and sleep and thus weakens the sleep drive. Behavioral changes like extending wakefulness in bed, in contrast to what the individual intends, can perpetuate insomnia into the chronic phase. Similarly, the patient might start having thoughts related to their sleep and recent injury such as “Lack of sleep will negatively impact my recovery,” or “My injury permanently damaged my ability to sleep well” which in turn increase hyperarousal, rumination and worry. This makes achieving restful sleep even more difficult. This collection of perpetuating behaviors and thoughts thus becomes the target on which CBT-I intervenes (46). For an individual with a mTBI who may not receive much assistance with sleep following the injury (aside recommendations to “get enough sleep,”) CBT-I could be a crucial intervention which changes the course of insomnia and thus recovery from mTBI. Although it is beyond the scope of this article to recommend specific adaptations of CBT-I in the context of mTBI, Ouellet et al. (47) recently published a book which comprehensively addresses this topic for providers.

## Potential Impacts of CBT-I on mTBI

One domain in which CBT-I could be particularly impactful for mTBI patients is cognitive functioning. A recent meta-analysis (48) demonstrated insomnia is associated with small to moderate deficits in cognitive functioning across numerous domains, including subjective cognitive performance and objective assessments of perceptual functioning, working memory (i.e., retention/capacity), episodic memory, complex attention, alertness, and executive functioning (i.e., problem solving). Each of these impairments could impact a patient's ability to engage in mTBI rehabilitation activities (e.g., following directions, completing out-of-session tasks, attending appointments) and thus reducing the burden of insomnia on cognitive functioning could improve engagement (49). Some existing evidence in populations without TBI suggests treating insomnia via CBT-I or other behavioral interventions may improve subjectively-assessed cognitive functioning (50), and preliminary evidence suggests some effect for objectively-assessed cognitive functioning (51, 52). However, more work is needed to understand the potential for improving cognitive functioning for patients with mTBI via CBT-I.

Another domain in which insomnia treatment could impact symptoms and rehabilitation course in mTBI is the improvement of psychosocial functioning (2). Unlike other forms of cognitive-behavioral therapy, CBT-I has a specific focus on sleep problems and in a manualized form does not directly treat other mental health symptoms. Nevertheless, a growing literature suggests treating insomnia in clinical populations is associated with significant improvements in pain (53), depression (54), anxiety (55), and posttraumatic stress symptoms (56). Each of these problems co-occurring with mTBI could markedly interfere with engagement in rehabilitation efforts or delay recovery (2). However, psychological treatment for each of these problems typically takes longer and may be less appealing to patients due to stigma or avoidance compared to CBT-I. Insomnia may be a more “approachable” treatment target than other psychosocial problems as it specifically targets sleep problems which may be less stigmatized than depression, anxiety, pain, and posttraumatic stress, yet CBT-I can also result in improvement across these domains in a relatively short time frame (57).

## Existing and Ongoing Research on Treatment of Insomnia and Sleep Problems in mTBI

### Research on CBT-I in Mild to Severe TBI

Despite the promise of CBT-I for patients with mTBI, previous work is limited (58). CBT-I has not been sufficiently examined in this population and there are no targeted treatments for TBI-related insomnia (59). In 2007, Ouellet et al. conducted a single-case experimental study of 8 weeks of CBT-I for 11 patients with mild to severe TBI and insomnia which developed following the TBI. The results demonstrated a significant improvement in sleep efficiency (i.e., the percentage of time in bed spent asleep) from pretreatment to posttreatment and 3-month follow-up (60). This study provides some preliminary evidence CBT-I may be useful in this population but is limited by small sample size and inclusion of a range of TBI severities.

### Research on Non-CBT-I Sleep Interventions in Mild to Severe TBI

Although studies of CBT-I for patients with TBI are scarce, some studies of treating related sleep problems in TBI can provide additional preliminary support. One study randomly assigned 24 adults with mild to severe TBI and complaints of sleep problems or fatigue to either treatment as usual or an 8-session cognitive-behavioral therapy (CBT) intervention for sleep and fatigue that included some elements of CBT-I. The results suggested the participants who received the CBT intervention demonstrated improvements in sleep quality, fatigue, and depression (61). Similarly, a pilot randomized controlled trial randomly assigned 24 adults with mild to moderate TBI and sleep difficulties to either a psychoeducation control condition or a 6-sesssion online CBT intervention that included some elements of CBT-I. Results suggested participants in the intervention condition experienced improvements in sleep disturbances but there was no effect for cognitive functioning or post-concussive symptoms (62). Finally, a prospective longitudinal study delivered individualized sleep recommendations (including sleep hygiene, medication, or sleep apnea treatment) to twelve adults with mild to severe TBI and found significant improvements in insomnia severity, depression severity, language, and speed of language processing (63). These studies provide some support for the impact of improving sleep in mild TBI but share significant limitations including heterogeneous TBI severity and sleep complaints, use of broad interventions, and small sample sizes. Further work examining targeted treatment (i.e., CBT-I) for diagnosed insomnia for individuals with mTBI will enhance understanding of the potential effect of CBT-I on the pervasive symptoms of mTBI.

### Ongoing Research on CBT-I in mTBI

Our group is currently conducting the first large randomized controlled trial of CBT-I in patients with mTBI (ClinicalTrials.gov identifier: NCT03261674). In this study, we will randomize 120 Veterans with mTBI to receiving either CBT-I or another behavioral treatment for insomnia and examine the effect of treatment on insomnia symptoms, quality of life, and functional outcomes. We will also explore potential mediators and moderators of response including cognitive arousal, initial sleep characteristics, posttraumatic stress symptoms, pain, chronic stress and inflammation. This study will help to fill the substantial gap in the literature of insomnia treatment in patients with mTBI.

## Current Challenges in using CBT-I in mTBI

More work examining the use of CBT-I in patients with mTBI is needed because they may present unique challenges for insomnia treatment compared to other populations. We have summarized some of the potential challenges here:

1. **Patients with mTBI struggle with attention, concentration, mental fatigue, or forgetfulness which may complicate the implementation of behavioral changes at home**. CBT-I relies on the patient to adhere to a set schedule, make changes to their bedtime routine, and complete daily sleep logs. Poor adherence to these guidelines may reduce the efficacy of the intervention or require some adaptation of CBT-I materials (e.g., additional reminders, simplified handouts, provisions for coping with fatigue, enlistment of a family member for support), although to date research has not been conducted to explore the efficacy of such modifications. However, one recent study of CBT-I for patients with mild cognitive impairment (64) resulted in significant improvements in sleep and cognitive functioning, suggesting CBT-I can be efficacious for individuals with memory impairment.
2. **Insomnia may co-occur with other sleep disturbances or disorders for which CBT-I may not be effective**. For example, mTBI is frequently associated with increased rates of daytime fatigue, obstructive sleep apnea, sleep fragmentation, or circadian rhythm disturbances (65). Although CBT-I can be safely used for insomnia in the context of these co-occurring sleep-wake disturbances, the benefit of CBT-I may be attenuated because symptoms from these disorders are unlikely to remit without targeted treatment. In other words, even if the insomnia is successfully treated, other untreated sleep disturbances may remain and cause continued impairment. In particular, patients with mTBI are more likely to experience daytime sleepiness (66) and severe fatigue (67). Daytime sleepiness is uncommon in a “typical” course of insomnia (i.e., people with insomnia without mTBI) and is not directly addressed by CBT-I. Although fatigue is common in insomnia, mTBI-related fatigue may not be fully alleviated by the typical strategies of CBT-I and thus require additional management. Both daytime sleepiness and fatigue may require the addition of alertness-promoting strategies to increase safety and maximize functioning and adherence to CBT-I. Similarly, the presence of circadian rhythm disturbances in mTBI may require specific attention that would typically be outside the scope of CBT-I but which could be successfully addressed by the addition of treatment components [e.g., light therapy; (68)].
3. **Individuals with mTBI who return to their typical activities are at increased risk for sustaining another head injury which may reduce the appropriateness of CBT-I**. In sustaining another head injury, the course of CBT-I may be disrupted or set back. For example, during the acute phase of injury, which can be associated with increased need for sleep rather than insomnia, the recommendations of CBT-I are unlikely to be beneficial and may require a delay in insomnia treatment. Little is known about the optimal time course of delivering CBT-I in the context of mTBI.

Given these considerations, more work is needed to understand the potential benefit of both unmodified and adapted CBT-I for patients with mTBI.

## Future Directions

There is a clear need for further research examining the potential utility of treating insomnia to improve the symptoms, recovery and rehabilitation of individuals with mTBI. Below, we have outlined priorities for future research in this area.

1. **Improvement of screening and assessment of sleep disorders in both clinical and research settings within the context of mTBI at all phases of injury using a validated and comprehensive measurement strategy [i.e., both subjective and objective sleep assessment;** **(****59****)] is vital**. Screening for sleep disorders should be standard practice for individuals with mTBI. This will contribute to a better understanding of the nature and course of sleep disturbances in mTBI and inform treatment development. Similarly, it is crucial to increase the integration of sleep services within the mTBI treatment context and develop the evidence base for targeted sleep-related recommendations and interventions. Detailed information about how to conduct a thorough assessment for sleep disorders among individuals with mTBI is presented in a recent book by Ouellet et al. (47).
2. **More clinical trials are needed which examine the impact of cognitive-behavioral interventions for sleep disturbances in mTBI**. As discussed above, our group has an ongoing trial to contribute to the evidence based for CBT-I among military veterans with mTBI. Additional studies are required to establish the efficacy and effectiveness of CBT-I or related interventions among additional subpopulations including children and adolescents, emerging adults, older adults, athletes, and mTBI comorbid to psychiatric or medical issues. We might expect that the variable presentation of mTBI among these subgroups could impact the delivery of CBT-I. For example, athletes may be focused on return to play or maximizing performance, which could change the focus of cognitive therapy. Alternatively, individuals with comorbid mental health diagnoses may benefit from the addition of cognitive-behavioral interventions which are not necessarily standard in CBT-I, such as behavioral activation. Once the efficacy of well-tested interventions (e.g., CBT-I) for specific sleep disorders is established in the mTBI population, adaptations that meet the specific needs of individuals with mTBI, and specific subgroups within the mTBI umbrella, will likely maximize benefits. For additional recommendations regarding specific directions for research on sleep and CBT-I in the context of mTBI, see a research agenda proposed by Wickwire et al. (68) and a recent review by Ford et al. (69).
3. **In addition to adaptations of CBT-I, the efficacy of other cognitive-behavioral interventions for sleep disorders should be explored for individuals with mTBI**. Transdiagnostic sleep interventions [e.g., TranS-C; (70)] that address sleep health may be useful to treat broad and co-occurring sleep disturbances. Although transdiagnostic sleep interventions have a smaller evidence base than CBT-I, they are an important direction of future research given the constellation of overlapping sleep complaints in mTBI. Similarly, acceptance-based nonpharmacological treatment of insomnia [e.g., mindfulness-based therapy for insomnia; (71)] might fit particularly well with the presentation of patients with CBT-I who may be coping with substantial and enduring changes to health and functioning. However, these interventions have yet to be tested among individuals with mTBI.
4. **Additional research is needed to understand the optimal timing of CBT-I delivery, potential modifications or adaptations, and which subpopulations are most likely to benefit**. Little is known about the ideal timing of the delivery of CBT-I to improve outcomes in mTBI. For example, should CBT-I be initiated immediately following the acute phase, or later on in recovery? What “dose” of CBT-I is optimal (e.g., typical dose of 6–8 sessions, or is a longer course required?) Can prevention strategies be put in to place early in the post-injury phase to minimize the development of poor sleep outcomes? As mentioned previously, adaptations may be needed to optimize CBT-I for TBI populations. Relatedly, little is known about which subpopulations of TBI patients (e.g., severity, co-occurring conditions) can be appropriately and effectively treated with CBT-I. This is a distal future direction, if initial work exploring the efficacy of CBT-I in the context of TBI demonstrates a promising signal.
5. **Further research is needed to explore the immediate and long-term effects of TBI sustained in childhood and young adulthood**. Most existing treatment research has focused on TBI sustained in adulthood, despite compelling evidence that sustaining TBI in childhood can increase the risk of sleep disturbances as long as 20 years after the event (72).
6. **It is important to consider the potential broader impact of CBT-I in the context of mTBI, particularly with regard to co-occurring conditions**. Nonpharmacological sleep interventions like CBT-I may indirectly improve symptoms of common co-occurring psychosocial problems (e.g., depression, chronic pain, posttraumatic stress) in mTBI. Another important area of exploration is how access to CBT-I might be linked to more distal outcomes including return to work or other meaningful activities, social engagement, and health-related quality of life. Along these lines, it is important to explore how the effective treatment of insomnia might change the course or evolution of cognitive and other mTBI-related symptoms.
7. **More research is needed to explore barriers to dissemination and implementation of sleep-focused interventions at the systemic level and design targeted programs to address these barriers**. It is especially important to consider leveraging existing resources to increase the dissemination of CBT-I (e.g., telehealth, online-delivered interventions) in populations with individualized needs. Increased access to training in CBT-I and sleep assessment, as well as availability of assessment and treatment tools are both crucial strategies that can help overcome the current provider-level barriers which limit access for individuals with CBT-I.

## Conclusions

In sum, sleep disturbances like insomnia are a novel, modifiable treatment target which may have a broad positive impact on the symptoms and recovery following mTBI. Thus, treating insomnia in individuals with mTBI could be a priority for clinicians and researchers alike. In this context, behavioral sleep medicine interventions like CBT-I should be seriously considered as they typically do not add to medication burden, have a strong existing evidence base, and may be more acceptable to patients than medication (57). CBT-I is well-accepted as an effective treatment for insomnia in many populations and co-occurring conditions, but it has not yet been adequately tested in the context of TBI and specific challenges remain. Thus, more research is needed to address screening and assessment procedures of sleep problems in TBI, efficacy, limitations, and benefits of CBT-I in TBI, and individualized factors which may affect the delivery and utility of CBT-I in TBI.

## Author Contributions

JD and AF both contributed to the planning and writing of this manuscript. All authors contributed to the article and approved the submitted version.

## Conflict of Interest

The authors declare that the research was conducted in the absence of any commercial or financial relationships that could be construed as a potential conflict of interest. The handling editor is currently organizing a Research Topic with one of the authors AF.
